# Effects of EpCAM overexpression on human breast cancer cell lines

**DOI:** 10.1186/1471-2407-11-45

**Published:** 2011-01-31

**Authors:** Johanna M Gostner, Dominic Fong, Oliver A Wrulich, Florian Lehne, Marion Zitt, Martin Hermann, Sylvia Krobitsch, Agnieszka Martowicz, Guenther Gastl, Gilbert Spizzo

**Affiliations:** 1 Laboratory for Experimental Oncology, Tyrolean Cancer Research Institute, Innrain 66, 6020 Innsbruck Austria; 2 Department of Haematology and Oncology, Innsbruck Medical University Anichstrasse 35, 6020 Innsbruck, Austria; 3 Division of Medical Biochemistry, Biocenter Innsbruck Fritz-Pregl Strasse 3, 6020 Innsbruck, Austria; 4 KMT-Laboratory Innrain 66, 6020 Innsbruck, Austria; 5 Max Planck Institute for Molecular Genetics, Ihnestrasse 73, 14195 Berlin, Germany; 6 Department of Haematology and Oncology, Franz Tappeiner Hospital, Via Rossini 5, 39012 Merano, Italy

## Abstract

**Background:**

Recently, EpCAM has attracted major interest as a target for antibody- and vaccine-based cancer immunotherapies. In breast cancer, the EpCAM antigen is overexpressed in 30-40% of all cases and this increased expression correlates with poor prognosis. The use of EpCAM-specific monoclonal antibodies is a promising treatment approach in these patients.

**Methods:**

In order to explore molecular changes following EpCAM overexpression, we investigated changes of the transcriptome upon EpCAM gene expression in commercially available human breast cancer cells lines Hs578T and MDA-MB-231. To assess cell proliferation, a tetrazolium salt based assay was performed. A TCF/LEF Reporter Kit was used to measure the transcriptional activity of the Wnt/β-catenin pathway. To evaluate the accumulation of β-catenin in the nucleus, a subcellular fractionation assay was performed.

**Results:**

For the first time we could show that expression profiling data of EpCAM transfected cell lines Hs578T^EpCAM ^and MDA-MB-231^EpCAM ^indicate an association of EpCAM overexpression with the downregulation of the Wnt signaling inhibitors SFRP1 and TCF7L2. Confirmation of increased Wnt signaling was provided by a TCF/LEF reporter kit and by the finding of the nuclear accumulation of ß-catenin for MDA-MB-231^EpCAM ^but not Hs578T^EpCAM ^cells. In Hs578T cells, an increase of proliferation and chemosensitivity to Docetaxel was associated with EpCAM overexpression.

**Conclusions:**

These data show a cell type dependent modification of Wnt signaling components after EpCAM overexpression in breast cancer cell lines, which results in marginal functional changes. Further investigations on the interaction of EpCAM with SFRP1 and TCF7L2 and on additional factors, which may be causal for changes upon EpCAM overexpression, will help to characterize unique molecular properties of EpCAM-positive breast cancer cells.

## Background

EpCAM is frequently overexpressed in human invasive breast cancer [1]. We reported EpCAM overexpression to be an independent prognostic marker for poor overall survival in node-positive breast cancer [2,3]. An independent group confirmed this finding in node-negative patients too [4]. Moreover, the magnitude of EpCAM antigen expression semiquantitatively assessed by immunohistochemistry showed a dose-dependent relationship with survival. In this retrospective analysis the patient subgroup with breast carcinomas overexpressing both EpCAM and Her-2/neu had the worst prognosis [5]. Targeting EpCAM with a humanized monoclonal antibody (Adecatumumab) in a randomized multi-centre phase II trial for the treatment of metastatic breast cancer yielded an expression- and dose-dependent reduction in formation of new metastatic lesions [6]. Recently, a trifunctional anti-EpCAM antibody (Catumaxomab) has received approval by the European Commission for the treatment of patients with EpCAM positive tumours [7].

EpCAM has initially been identified as a cell adhesion molecule located within intercellular adherens junctions, where it modulates cadherin-mediated cell adhesion and promotes epithelial cell migration and proliferation. EpCAM expression is not only involved in epithelium formation and epithelial-mesenchymal transition during organ development and tissue repair but also contributes to epithelial cell transformation [8,9]. Regarding EpCAM target genes, overexpression of EpCAM was found to be associated with enhanced transcription and translation of the proto-oncogene c-myc and the cell cycle proteins cyclin A and E in human epithelial 293 cells as well as in murine NIH3T3 fibroblasts [10]. Furthermore, proteome analysis revealed the epidermal fatty acid binding protein E-FABP, a major target of c-myc, to be upregulated upon EpCAM expression in HEK293 cells. Enhanced E-FABP expression correlated with EpCAM expression levels in squamous cell carcinoma lines and in primary head and neck carcinomas [11]. Very recently, the proteolytic shedding of the intracellular domain of EpCAM (EpICD) was shown to confer a mitogenic signal, participating in a multimeric nuclear complex together with FHL2, β-catenin and Lef-1 for the induction of target gene transcription in FaDu hypopharynx and HCT-8 colon carcinoma cells [12,13]. Furthermore, our group described that DNA methylation is a potential mechanism for the regulation of EpCAM expression [14].

Knowledge on the role of EpCAM in the process of carcinogenesis, tumour progression and metastasis needs further elucidation. Presumably, consequences of EpCAM overexpression and signaling may strongly depend on the tumour type, stage and the tumour microenvironment. This assumption is corroborated by the simple clinical observation that the prognostic impact of EpCAM expression depends on tumour type, disease stage and host antitumour immunity [12,15]. Contradictory findings from various cell culture systems support the view that EpCAM expression can modulate cell proliferation, differentiation and migration, but the outcome of modulation is strongly dependent on cell type and origin [16-18].

So far little data exist on EpCAM signaling in breast cancer. The impact of EpCAM expression in human breast cancer cell lines was investigated in loss-of-function studies by silencing EpCAM expression in EpCAM-positive breast cancer cell lines, which resulted in a decrease in cell proliferation, migration and invasiveness, with a concurrent increase of the detergent-insoluble protein fractions of E-cadherin and α- and β-catenin. Importantly, those observations could be confirmed only partially with the weakly EpCAM-positive non tumourigenic breast cancer cell line MCF-10A [17]. Since EpCAM signaling and function has been studied primarily in EpCAM-positive breast cancer cell lines with siRNA-based knockdown, we aimed to generate overexpression breast cancer cell lines and characterize these cell line models by analysing migration, proliferation and transcriptional changes.

## Methods

### Cell lines and plasmids

All cell lines were obtained from American Type Culture Collection. Hs578T, MDA-MB-231 and MCF-7 cell lines were grown in phenol red free MEM medium (PAA) supplemented with 5 mM glutamine (Gibco) and 10% fetal bovine serum (FBS superior, Biochrom) at 37°C under 5% CO^2^. SKBr-3 cells were cultivated in McCOYs (Gibco) substituted with 5 mM glutamine and 10% FBS. A mixture of proteolytic and collagenolytic enzymes (Accutase, PAA) was used for detachment of the adherent cells for passaging. All cell lines were cultivated at maximum to passage 25 (3 months) and the absence of mycoplams contamination was controlled by using Venorgem PCR based detection kit (Minerva Biolabs).

EpCAM coding sequence was cloned into the pIRESpuro3 vector (Clontech) to get a high CMV promoter driven protein expression. Stable EpCAM expressing cell lines as well as respective empty vector control lines were established by transfections using Nucleofector Kit V (Amaxa) followed by puromycine selection.

### Western blotting

50 μg of protein heated in Laemmli sample buffer was loaded per lane, resolved by a 4-20% SDS-PAGE (Criterion, Biorad) and transferred onto a nitrocellulose membrane (0.2 μm pore size, Schleicher & Schuell) for western blotting at 150 mA for 2 hours. To control protein transfer, the membrane was incubated 5 min in a Ponceau S staining solution until protein lanes were visible. After washing steps with TBS-T, the membrane was blocked 1 hour with 10% non-fat dry milk diluted in TBS-T. Primary antibodies were incubated in 1% non-fat dry milk diluted in TBS-T at 4°C over night. Secondary HRP-labelled antibodies were incubated for 1 hour at room temperature. After incubation with ECL Western blot detection reagent (Amersham), the detection followed by exposing the membrane to an X-ray film (Agfa).

Antibodies used for Western analysis were: C-10 (mouse monoclonal against amino acids 24-93 of human EpCAM, Santa Cruz Biotechnology), E144 (rabbit monoclonal antibody against the C-terminus of human EpCAM, Epitomics), β-Catenin (BD Biosciences), pan-actin (Ab-5 Clone, ACTN05, Neomarkers), GAPDH 6C5 (Santa Cruz Biotechnology), lamin A/C (2032, Cell Signalling) and anti-TCF7L2 (clone 6H5-3, Upstate Biotechnology).

### Immunofluorescence

Cells were plated at different densities on non-coated or gelatine-coated glass coverslips and prepared for immunofluorescence after additional 48 h hours. Cells were washed 3 times in PBS, extracted in 2% PFA with 0.3% Triton X-100 in PBS for 5 minutes and fixed in 2% PFA in PBS for 30 minutes. Primary antibodies (NCL-ESA, 1:100, mouse monoclonal anti-EpCAM, clone VU1D9, Novocastra and C-10, 1:50, Santa Cruz Biotechnology) were used with the respective Alexa 488 labelled secondary serum (Molecular probes). F-actin was visualized using Alexa 568 Phallodin (Molecular probes).

Confocal microscopy was performed with a microlens-enhanced Nipkow disk-based confocal system UltraVIEW RS (Perkin Elmer, Wellesey MA, USA) mounted on an Olympus IX-70 inverse microscope (Olympus, Nagano, Japan). Images were aquired using the UltraVIEW RS software (Perkin Elmer).

### Subcellular fractionation

To generate nuclear and cytoplasmic lysates NE-PER fractionation kit was used (Pierce 78833). A stepwise lysis of cells generated both functional cytoplasmic and nuclear protein extracts. Protocol was performed according manufacturer's instructions.

### Cell counting kit

Proliferation and cell viability analysis was performed by using Cell Counting Kit-8 (CCK-8, Dojindo Molecular Technology) according manufacturer's instructions. Cells plated in 96-well plates (5 replica wells for each cell line). At intervals of 24 hours, CCK-8 solution was added directly to the cells (dilution 1:10) and the absorbance at 450 nm was measured after 4 hours of incubation in a microplate reader (Biorad). Cell numbers of non transfected cells have been quantified once in parallel by thymidine incorporation assay. Different clones of transfected cell lines were analysed.

### Chemotherapeutic sensitivity testing

Cells were seeded at equal amounts (1000 cells for MDA-MB-231, 5000 cells for Hs578T) into 96 well plates and allowed to adhere over night. Then, cells were treated for 24 hours with different doses of Docetaxel (Taxotere^®^, Sanofi Aventis): respectively Hs578T - 0, 0.005, 0.01, 0.1, 0.5 μg/mL; MDA-MB-231 - 0, 0.01, 0.1, 0.5, 1 μg/mL. CCK-8 solution was added to determine cell viability and proliferation. Absorbance was measured at 450 nm after 4 hours of incubation.

### Matrigel invasion assay

The FluoroBlok BioCoat™ Tumour Invasion System (BD Biosciences) was used to assess the invasive potential of cell lines *in vitro*. Membranes (8 μm FluoroBlok™ PET membranes coated with a uniform layer of BD Matrigel™ matrix) were rehydrated and cells were seeded into the upper chamber insert. To facilitate migration and invasion a serum gradient was used. Cells were allowed to migrate for 22 hours. To quantify migration, cells in the lower chamber were stained with calcein (BD), and the fluorescence was measured at Cytofluor4000 fluorescence reader (MTX Lab Sytems). The amout of migrated cells in the EpCAM transfected lines was compared to the migration rates of their respective empty vector control lines.

### Wnt signal activity determination

The Cignal™ TCF/LEF Reporter Kit (SABiosciences, MD) was used to determine Wnt signaling activity. A construct containing a promoter with TCF binding motifs in front of a *Firefly *luciferase gene was transfected into the cell lines. β-catenin binding to the TCF motif resulted in the transcription and translation of the luciferase and in the emission of a bioluminescence signal which was detected on a CHAMELEON multitechnology platereader (Hidex). To normalize on transfection efficiency, a CMV promoter driven *Renilla *luciferase was co-transfected. The luciferase substrates were purchased from Promega (Dual-Luciferase^® ^Reporter (DLR) Assay, E1910) and added automatically by a dual-injector system according to manufacturer's protocol.

Each cell line was transfected in triplicates with the TCF construct (or negative/positive control construct containing manipulated TCF binding motifs) plus the *Renilla *control using SUREfect transfection reagent (SABiosciences) and assayed after 48 hours. Bioluminescence signal intensity was determined after normalization on transfection intensity according to following formula: Relative Response Ratio = (Signal_Repoter_-Signal_Negative Control_)/(Signal_Positive Control_-Signal_Negative Control_)

### RNA isolation and analysis

Total RNA was isolated by using TRI-reagent (Molecular Research Centre) alone or in combination with the RNeasy MinElute Cleanup Kit (Qiagen) following the manufacturer's protocol. Concentration and purity of each sample were assessed by absorbance at 260 nm and by the 260/280 nm ratio, respectively. Phenol contaminations were controlled by the 260/270 nm ratio and the concentration was adjusted [19]. The integrity of the RNA was controlled on ethidium bromide-stained agarose-formaldehyde gels. For the samples used for Affimetix Chip analysis, RNA quality was determined on an Agilent 2100 Bioanalyzer and by photometric analysis.

### Array data analysis

GC Robust Multi-array Average (GCRMA) background adjustment, quantile normalization and median-polish summarization on Affymetrix HGU133 plus 2.0 microarray probe-level data were performed using R 2.7.1 and Bioconductor 2.2. (gcrma package: justGCRMA function). The log2 transformed absolute expression values of the treatment sample (EpCAM positive line) were subtracted from the absolute expression values of the control sample (control line) in a gene-wise manner. The resulting log2 transformed relative expression values were ranked according to the level of the values. The top 5% and lowest 5% of the probesets were selected for further analysis. Ingenuitiy^® ^Pathway Analysis (IPA) software (Ingenuity Systems Inc., Redwood City, CA) was used to inter-connect GO classified categories of the obtained gene set in a context specific manner in order to establish a regulatory network model.

### Real-time PCR

Single stranded cDNA was synthesized from 1 μg total RNA using random primers and SuperScript II Reverse Transcriptase (Invitrogen) according manufacturer's instructions. Real-time analysis was performed using 20 ng single stranded cDNA as well as specific primers and SensiMixPlus containing SYBR^® ^Green (Quantace) on a Rotor Gene 6000 cycler (Corbett Research) using following conditions: 95°C 300 sec; 40 cycles: 95°C 10 sec, 60°C 15 sec, 72°C 10 sec (acquiring on Sybr Green). Primer specificity was checked by melting curve analysis. For each primer pair (Table 1) the PCR amplicon length was verified once by gel electrophoresis and the sequence was analysed. All applications of different cDNA input (n = 3, n = 4) were performed in replicates. The TATA box-binding protein (TBP) was used as endogenous control for normalization [20,21].

**Table 1:** Primer sequences used for real-time PCR

Gene	GenBank Acc. Nr.	sequence (5'to 3')
TBP	NM_003194	fwd TGCACAGGAGCCAAGAGTGAA
		rev CACATCACAGCTCCCCACCA
		
EpCAM	NM_002354	fwd CGCAGCTCAGGAAGAATGTG
		rev TGAAGTACACTGGCATTGACG
		
TCF7L2	NM_030756	fwd TGCACTGTCCAGAGAAGAGC
		rev GCTGCTTGTCCCTTTTCCTC
		fwd TCAATGAATCAGAAACGAATCAA
		rev CTCTTGGCCGCTTCTTCC
		
SFRP1	NM_003012	fwd GAGTTTGCACTGAGGATGAAAA
		rev GCTTCTTCTTCTTGGGGACA
		
ITF-2	NM_001083962	fwd AGCCATTCTCTTCTGCCAAA
	NM_003199	rev CAGGTTCTCATCACCCTCGT
CCND1	NM_053056	fwd GCTGCTCCTGGTGAACAAGC
	NM_001758	rev TTCAATGAAATCGTGCGGG
		
MYC	NM_002467	fwd CTACGCAGCGCCTCCCTCCACT
		rev GGCGCTCCAAGACGTTGTGTGTTC

Relative expression ratios (R) of target genes were calculated based on the normalized Ct deviation of the EpCAM expressing cells versus the control cells according the mathematical model described by M. Pfaffl: ratio = (2^∆Ct_target_(control-EpCAM))/(2^∆Ct_TBP_(control-EpCAM)). The Ct value is defined by the cycle at which the threshold is crossed, and ∆Ct is the crossing point difference between sample and control [22].

The relative expression software tool REST 2008 (by M. Pfaffl and Corbett Research) was used for statistical analysis. Two groups (controls and EpCAM-positive cells) were compared based on their mean crossing point deviation for significance by a randomization test. The resulting hypothesis test value *p*(H1) is an indicator of probability that the difference between sample and control group is significant [23,24].

## Results

Only few commercially available immortalized human breast cancer cell lines lack EpCAM expression. Hs578T and MDA-MB-231 cells were found to express none or only very little EpCAM mRNA and protein in comparison to established and well characterized breast cancer cell lines such as MCF-7 or SK-BR-3 [25].

Both Hs578T and MDA-MB-231 cells were stably transfected with the EpCAM cDNA containing construct pIRESpuro3_EpCAM. The resulting cell lines were designated Hs578T^EpCAM ^and MDA-MB-231^EpCAM^; the cell lines transfected with the empty control vector pIRESpuro3 were named Hs578T^control ^and MDA-MB-231^control^, respectively. Parental MDA-MB-231 cells were originally derived from a malignant pleural effusion. MDA-MB-231 cells are highly proliferative, invasive *in vitro *and tumourigenic in nude mice [26]. Hs578T cells were derived from an invasive ductal carcinoma, show low invasive potential and are non-tumourigenic [27,28]. Of note, both cell lines lack E-cadherin expression (due to promoter methylation), which is a common feature among carcinoma cell lines with low or absent EpCAM expression.

### EpCAM protein undergoes posttranslational modification

**Figure 1:**
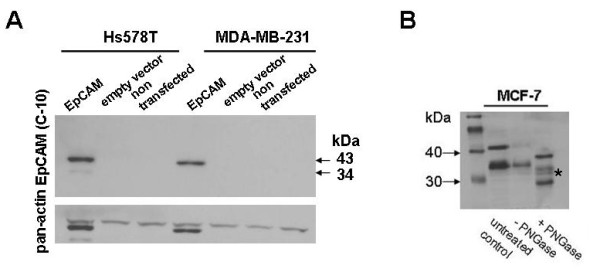
**EpCAM protein expression**. (A) Western blot analysis of EpCAM in the stable transfectants and control cell lines. Observation of the appearance of two protein species (43 and 34 kDa). Actin was used as loading control. Its molecular weight is about 45 kDa. (B) PNGase F treatment of MCF-7 cells. To analyse if the two EpCAM protein species derive from distinct glycosilations, MCF-7 lysates were incubated with the glycosidase PNGase F. As both protein bands shift in height, N-linked glycosilation is not responsible for the existence of two species (* = potential degradation products).

Using SDS-PAGE, EpCAM protein was detectable as a double band in both transfected breast cancer cell lines as well as in MCF-7 control lines under denaturing conditions. After treating cell lysates with the glycosidase PNGase F, both of these EpCAM protein bands were shifted to a lower molecular weight but were still detectable as a doublet, indicating that N-linked glycosilation is not responsible for these two different species of the EpCAM protein (Figure 1). However, this posttranslation modification might be of functional importance.

### Localization of EpCAM determined by confocal microscopy

**Figure 2:**
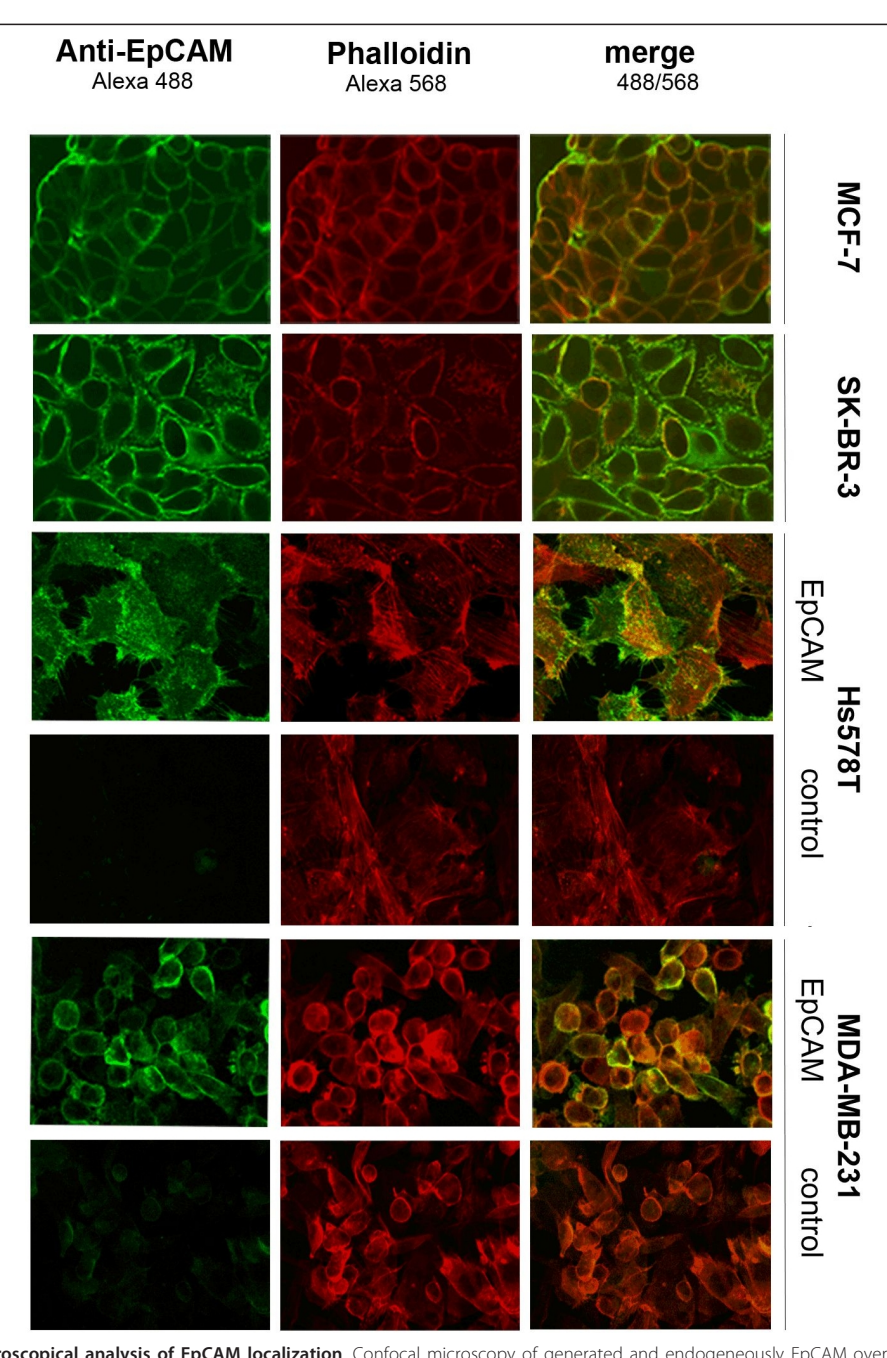
**Microscopical analysis of EpCAM localization**. Confocal microscopy of generated and endogeneously EpCAM overexpressing cell lines as well as vector controls revealed an increase of EpCAM membraneous staining with cell density. Cells with a cytosolic distribution could be observed in cultures of low confluence. Actin staining with phalloidin revealed slight changes of the actin cyctoskeleton formation upon EpCAM expression.

Confocal microscopy analysis revealed that localization of EpCAM antigen was strongly influenced by cell density in monolayer cell cultures. Highly confluent monolayers showed a predominantly membranous EpCAM staining, while single cells lacking contact with neighbouring cells exhibited much weaker membrane but stronger cytosolic staining. Cell membrane areas at intercellular contact regions stained strongest for EpCAM protein (Figure 2). Since physical interactions of EpCAM and E-cadherin have been proposed [9], confocal microscopy analysis demonstrated that E-cadherin expression at the cell surface was not necessary to recruit EpCAM to the cell membrane in confluent Hs578T^EpCAM ^and MDA-MB-231^EpCAM ^cells.

The results obtained by confocal microscopy and subcellular fractionation in the transfected EpCAM-positive cell lines were comparable to the endogenously EpCAM-expressing MCF-7 and SK-BR-3 cell lines. For this reason, Hs578T^EpCAM ^and MDA-MB-231^EpCAM ^cells provided us with an excellent tool to investigate EpCAM-mediated changes on cellular and molecular levels.

### Hs578T^EpCAM ^cells show enhanced proliferation and chemosensitivity

**Figure 3:**
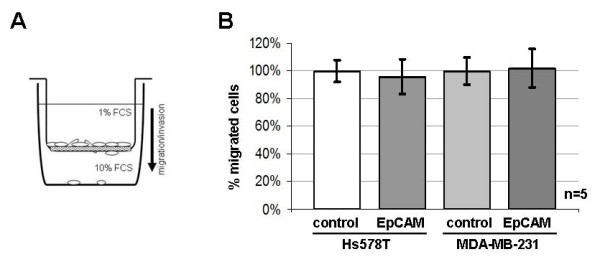
**Migration/invasion assay**. **(A) **Schematic presentation of an Fluoroblock BioCoat™ invasion chamber (BD) composed of a Matrigel™ coated transwell insert containing an 8 μm pore size membrane. The Matrigel matrix locks the pores of the membrane and therefore blocks non invasive cells from migrating. Invasive cells can degrade this basement membrane reconstitution and migrate through the pores. **(B) **Cells have been seeded at high density into the upper chamber. Migration was stimulated by a serum gradient. After 22 hours the amount of migrated cells has been measured with the Cytofluor4000 fluorescence reader (MTX Lab Systems) from the bottom after cell labelling with calcein (BD), a staining dye for vital cells only. The FluoroBlok™ membrane efficiently blocked the transmission of light into the upper chamber, therefore the measured signal derived from migrated cells only. Although all cells migrated efficiently, no difference could be detected by comparing Hs578T^EpCAM ^and MDA-MB-231^EpCAM ^cells with their respective empty vector control lines (set as 100%).

EpCAM overexpression has been reported to be associated with a strongly invasive and aggressive tumour phenotype in breast cancer [2,3,17]. However, by using a Matrigel™ coated Boyden chamber assay to compare the *in vitro *migration/invasion properties of EpCAM-expressing cell lines with their EpCAM-negative empty vector counterparts, no difference in cell migration and invasiveness could be found even by optimizing experimental conditions and prolonging migration time (Figure 3).

**Figure 4:**
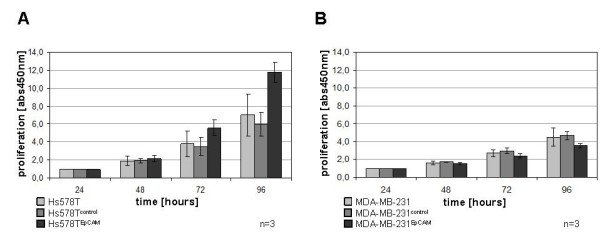
**Cell proliferation assay**. In a tetrazolium salt based assay, the proliferative behavior of cell lines with stable EpCAM expression in comparison to empty vector control and untransfected cells has been measured. EpCAM transfected Hs578T cells show enhanced doubling frequency than non-transfected cells and empty vector controls. This effect appears two days after cell seeding. No similar effect could be detected in the stable transfected MDA-MB-231^EpCAM ^cell line. Absorbance was normalized to the 24 hours values.

**Figure 5:**
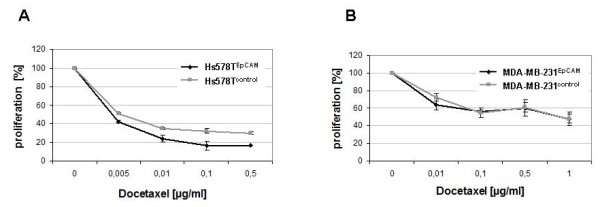
**Docetaxel sensitivity testing**. Hs578T^EpCAM ^and Hs578T^control ^cells **(A) **as well as MDA-MB-231^EpCAM ^and MDA-MB-231^control ^**(B) **cells have been treated one day after seeding with different amounts of the chemotherapeutic Docetaxel for 24 hours. Sensitivity to the chemotherapeutic was evaluated in % proliferation (±SEM) with 100% proliferation for untreated cells. Hs578T^EpCAM ^cells were more sensitive to Docetaxel treatment as compared to their EpCAM negative counterpart Hs578T^control^, whereas no difference in sensitivity could be detected in MDA-MB-231 transfectants. Docetaxel is known to inhibit mitosis by blocking the microtubule apparatus, therefore we suggest that the enhanced sensitivity of Hs578T^EpCAM ^cells can be related to their enhanced proliferation rate.

To assess cell proliferation, a tetrazolium salt based assay (CCK-8) was performed. The same methodology was used by other groups for the functional analysis of EpCAM function in EpCAM overexpressing HEK293 cancer cells, where EpCAM positive cells exhibited enhanced proliferative and metabolic activity compared to cells with low or no antigen expression [10,11,29]. We additionaly compared proliferation by thymidine incorporation with the results of the CCK-8 test, and found the CCK-8 assay to be a reasonable alternative to classical radioactive methods for these cell lines. In our experiments, Hs578T^EpCAM ^cells showed a shortened doubling time as compared to non transfected cells and empty vector controls. This effect has been observed 48 to 72 hours after cell seeding (Figure 4) and was reproduced in different transfected cell clones. However, this growth-promoting effect could not be detected by comparing MDA-MB-231^EpCAM ^cells to the respective empty vector control cells, suggesting that the *in vitro *growth-promoting effect of the EpCAM antigen is not universal but depends on individual cell features. In line with these results, Hs578T^EpCAM ^but not MDA-MB-231^EpCAM ^cells showed enhanced chemosensitivity to Docetaxel treatment compared to their empty vector counterparts (Figure 5).

### Hs578T^EpCAM ^and MDA-MB-231^EpCAM ^cells show expression changes of Wnt pathway components

**Figure 6:**
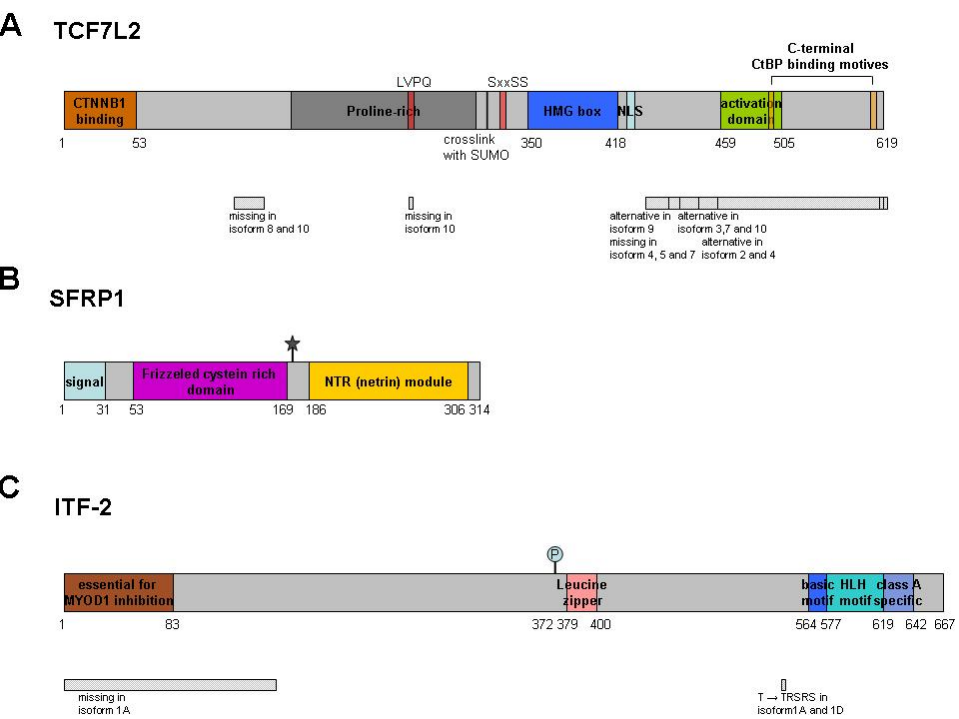
**Schematic representation of TCF7L2, SFRP1 and ITF-2**. **(A) TCF7L2** can be divided in 3 major domains: the β-catenin binding domain (CTNNB1), the DNA-binding HMG box and a C-terminal domain which is variable in many isoforms. Only the long form is able to bind to the COOH-terminal binding protein (CtPB), the C-terminus has an important regulatory function. The presence of the CtBP motif is required for the possible repressor function of TCF7L2. Many splicing variants (>16) have been described and each cell expresses more than one variant [32,37]. **(B) SFRP1 **is a secreted protein and contains a frizzled typical cysteine rich domain (Fz-CRD) which displays the putative Wnt-ligand binding site. The C-terminal part of the protein shows homology to netrin (NTR), an extracelluar protein involved in axonal guidance. NTR domains are involved in protein binding and contain segments rich of positively charged residues. This region is reported to bind to heparin [34]. **(C) ITF-2 **binds to DNA via its basic-helix-loop-helix (bHLH) domain and can be regulated by β-catenin. The Pitt-Hopkins syndrome (PTHS), a rare syndromic encephalopathy, is caused by ITF-2 haploinsufficency. At least 3 isoformal sequences have been described. The two major isoforms, ITF-2A and ITF-2B, have different N-termini resulting from alternative promoter usage [30]. (⋆ = glycosilation, p = phosphorylation. Swissprot accession numbers Q9NQB0, Q8N474, P15884)

In order to investigate changes of the transcriptome on EpCAM gene overexpression in human breast cancer cells, global gene expression analysis using the human genome U133 Plus 2.0 chip (Affymetrix) was performed. Differential gene expression analysis was performed by comparing expression levels from Hs578T^EpCAM ^cells (GEO accession numbers: GSE25743, GSM632607, GSM632608) with the respective empty vector control. The top and lowest 5% of probe sets were used for further analysis by Ingenuitiy^® ^Pathway Analysis (IPA) software (Ingenuity Systems Inc., Redwood City, CA). Interestingly, alterations of the Wnt signaling pathway components were found in both EpCAM-transfected cell lines. In more detail, we found the secreted frizzled related protein 1 (SFRP1) - a Wnt ligand competitor and therefore signaling inhibitor - and the HMG box containing transcription factor 7 like 2 (TCF7L2), a Wnt-responsive transcription factor which can also act as repressor of signaling, to be significantly downregulated after EpCAM overexpression. Additionally, the mRNA level for the immunoglobulin transcription factor 2 (ITF-2) was diminished. Only recently, ITF-2 has been identified as a Wnt downstream target [30]. A schematic representation of SFRP1, TCF7L2 and ITF-2 proteins is illustrated in Figure 6.

**Figure 7:**
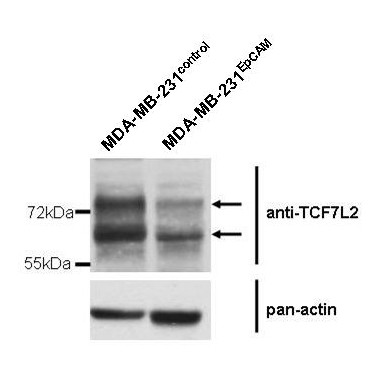
**TCF7L2 protein expression**. Western Blot showing lower amounts of TCF7L2 protein expression in MDA-MB-231^EpCAM ^cells compared to their empty vector counterparts.

Changes on the mRNA level detected by expression arrays were confirmed by real-time PCR. TATA box-binding protein (TBP) mRNA expression was not significantly different between control and EpCAM-positive cell lines and served as optimal housekeeping gene. To determine the expression differences of SFRP1, TCF7L2 and ITF-2 in EpCAM positive cells we calculated normalized mean expression levels (Table 2). SFRP1-specific mRNA was consistently downregulated up to 35.8-fold in Hs578T^EpCAM ^and up to 11-fold in MDA-MB-231^EpCAM ^cell lines in comparison to the respective controls. TCF7L2, was downregulated in MDA-MB-231^EpCAM ^cells with a 3.6-fold change while in Hs578T^EpCAM ^cells the TCF7L2 mRNA level was only marginally decreased. In line with these results, protein levels of TCF7L2 were downregulated in MDA-MB-231^EpCAM ^cells (Figure 7). ITF-2 mRNA was downregulated in MDA-MB-231^EpCAM ^cells until the limit of detection (>1500-fold, ΔΔCt value > 10). In Hs578T^EpCAM ^cells, ITF-2 expression was decreased only 3.5 times.

**Table 2:** Real-time PCR

	Expression of							
	TBP		SRFP1		TCF7L2		ITF-2	
n = 3	Ct ± SD	CV[%]	Ct ± SD	CV[%]	Ct ± SD	CV[%]	Ct ± SD	CV[%]
**MDA-MB-231**^**control**^	22.11 ± 1.90	4.95	**31.42 ± 2.90**	9.22	**19.19 ± 1.45**	7.54	**23.47 ± 0.43**	1.82
**MDA-MB-231**^**EpCAM**^	21.81 ± 0.10	0.47	**34.37 ± 1.80**	5.25	**20.75 ± 0.27**	1.33	**36.26 ± 5.50**	15.18
	**comparison EpCAM expressing versus control**							
**Regulation Factor**			**-11.06**		**-3.64**		**-1773.84**	
**REST analysis**								
**P(H1)**			0.000		0.000		0.000	
**Result**			**DOWN**		**DOWN**		**DOWN**	
								
	**Expression of**							
	**TBP**		**SRFP1**		**TCF7L2**		**ITF-2**	
n = 4	Ct ± SD	CV[%]	Ct ± SD	CV[%]	Ct ± SD	CV[%]	Ct ± SD	CV[%]
**Hs578T**^**control**^	23.23 ± 0.72	3.09	**25.35 ± 2.07**	8.17	21.28 ± 0.86	4.04	22.73 ± 1.81	7.96
**Hs578T**^**EpCAM**^	22.54 ± 0.18	0.81	**28.50 ± 3.39**	11.91	20.76 ± 0.56	2.71	23.42 ± 0.54	2.30
	**comparison EpCAM expressing versus control**							
**Regulation Factor**			**-35.82**		-0.73		-3.55	
**REST analysis**								
**P(H1)**			0.048		0.658		0.128	
**Result**			**DOWN**		not different to control		not different to control	

Quantification of differential mRNA expression of the Wnt signaling components SFRP1, TCF7L2 and ITF-2 in the stable EpCAM expressing cells in comparison to the empty vector control lines. (SD = standard deviation, CV = coefficient of variation, REST P(H1) = hypothesis test p value, significant if < 0.05).

In order to evaluate the obtained regulations in a group-wise comparison we tested the expression differences between EpCAM positive cell lines and the respective controls for significance with a Pair Wise Fixed Reallocation Randomization test by using the REST^© ^relative expression software tool. The perceived differences between EpCAM expressing cells and controls were of statistic significance for the downregulation of SFRP1 in both MDA-MB-231^EpCAM ^and Hs578T^EpCAM ^cell lines and for TCF7L2 and ITF-2 in MDA-MB-231^EpCAM ^cells (*p*(H1) < 0.05).

An EpCAM-associated downregulation of inhibitory and repressor molecule expression might contribute to the activation or enhancement of Wnt signaling in breast cancer and therefore further corroborate the ongogenic potential of the EpCAM tumour antigen.

### Nuclear accumulation of β-catenin in MDA-MB-231^EpCAM ^cells

**Figure 8:**
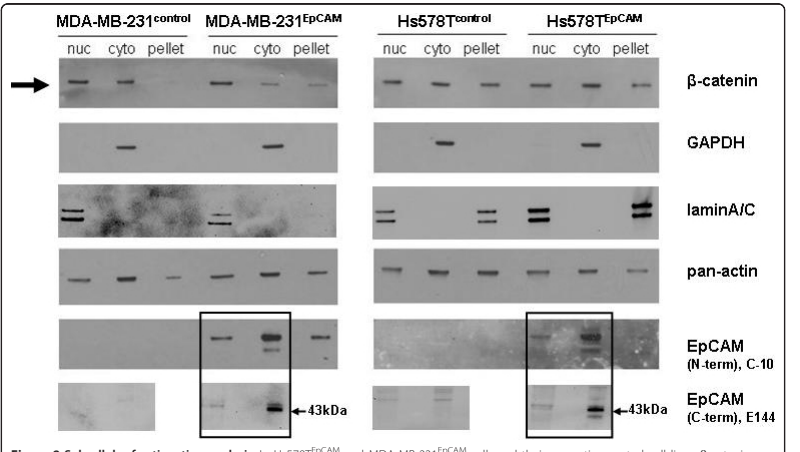
**Subcellular fractionation analysis**. In Hs578T^EpCAM ^and MDA-MB-231^EpCAM ^cells and their respective control cell lines, β-catenin was highly expressed in all cell lines, but only in the MDA-MB-231^EpCAM ^cell line a significant and reproducible accumulation of β-catenin could be observed in the nuclear compartment, which resulted in an increased Wnt signaling activity. The mainly cytosolic protein GAPDH was used to control the purity of the cytosolic fractionation. To show integrity of the nuclear fraction lamin a/c antibody was used. Pan-actin was used as loading control. As cells were cultivated to 70-80% density, a majority of the EpCAM protein was present in the cytosol. Additionally to the N-terminal directed anti-EpCAM antibody C-10, EpCAM was detected with a C-terminal directed antibody (E144). Interestingly, EpCAM could be detected in the nuclear fraction with both antibodies.

Fractionation analysis of cell lysates revealed the appearance of EpCAM protein in the soluble cytosolic, the nuclear and the insoluble membraneous fractions in the transfected Hs578T^EpCAM ^and MDA-MB-231^EpCAM ^cell lines (Figure 8). This confirmed the distribution pattern obtained by our group and others in breast cancer-derived as well as in mouse fibroblast cell lines [17,31]. In MDA-MB-231^control ^and in HS578T^EpCAM ^as well as in Hs578T^control ^cells, similar amounts of β-catenin were found in the cytosolic and in the nuclear protein fraction (Figure 8). In MDA-MB-231^EpCAM ^cells, a nuclear accumulation of ß-catenin was accompanied consistently by a decrease in β-catenin levels in the cytosol, whereas in Hs578T cells the expression of EpCAM had no significant impact on β-catenin distribution.

As cells were cultivated only to 70-80% density, a majority of the EpCAM protein (detected by C-10 antibody) was present in the cytosol. EpCAM was additionally detected with a C-terminal directed antibody (E144). The presence of EpCAM in the nuclear fraction suggests localization in the perinuclear compartment.

### Higher activity of Wnt pathway signaling in MDA-MB-231^EpCAM ^cells

**Figure 9:**
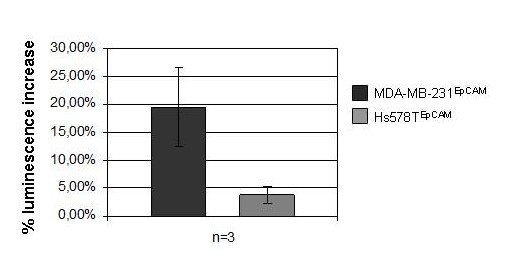
**Wnt signaling activity assay**. Upon activation of Wnt signaling a luciferase emitted bioluminescence signal is measured. A reporter plasmid containing a constitutive active TCF/LEF motif determined the 100% activation value while a mutated motif lacking any consensus site for β-catenin binding represented the background luminescence level. Hs578T^EpCAM ^cells showed only a modest activation, whereas EpCAM overexpression in MDA-MB-231^EpCAM ^could activate Wnt signaling about 20% in comparison to their empty vector controls.

To further confirm these data, the Cignal™ TCF/LEF Reporter Kit (SABiosciences, MD) was used to measure the transcriptional activity of a β-catenin-responsive luciferase reporter. After 48 hours of incubation luciferase activities were evaluated, normalized to the transfection controls and the signaling intensities of EpCAM positive cells were then compared with the values obtained from the corresponding control cell lines (Figure 9).

Wnt activity in Hs578T^EpCAM ^cells was only slightly higher than in Hs578T^control ^cells (3.7% +/- 1.4%) suggesting that down-regulation of the SFRP1 inhibitor alone is not sufficient to achieve a strong activation of Wnt signaling. In contrast, MDA-MB-231^EpCAM ^cells showed a 19.5% +/- 7.0% higher activity of Wnt pathway signaling compared to empty vector control cells.

## Discussion

Our results show the effect of constitutive EpCAM expression in previously EpCAM antigen negative or low expressing parental human breast cancer cell lines Hs578T and MDA-MB-231. Of note, most other commercially available human breast cancer cell lines are characterized by a high level of EpCAM protein expression, thus low or even no EpCAM expression is a rare occurring property. Hs578T cells are used as an example for a low-tumourigenic EpCAM-negative cell line, whereas MDA-MB-231 is used as an example for a more tumourigenic one. Given that only these two breast cancer cell lines could be used for gain-of-function studies, and the resulting phenotypes were not consistent, further investigation will be needed to elucidate a potential *in vivo *relevance of the presented findings. However, the expression of EpCAM affected cancer-related signaling molecules in both cell lines and seemed to contribute in triggering complex biological processes, which account for an aggressive tumoural behaviour.

In the transfected, constitutively EpCAM-expressing human breast cancer cell lines described here, we observed a reduced expression of Wnt signaling associated components after EpCAM overexpression. We found the downregulation of mRNA levels for SFRP1 in both Hs578T^EpCAM ^and MDA-MB-231^EpCAM ^cells, and TCF7L2 in MDA-MB-231^EpCAM ^only. These are two important negative regulators of Wnt signaling [32]. The results on proliferation and Wnt signaling activation between cell lines were not consistent. As such, Wnt signaling was activated significantly in MDA-MB-231^EpCAM ^but not in Hs578T^EpCAM^. In contrast to these data, proliferation and chemosensitivity was increased in Hs578T^EpCAM ^but not in MDA-MB-231^EpCAM^.

Canonical Wnt signaling hinges on four complexes: the Wnt-frizzled receptor complex in the membrane, the β-catenin destruction complex, the nuclear TCF/LEF/β-catenin transcriptional activator complex and the nuclear TCF/LEF transcriptional repressor complex [33]. SFRP1 acts as a repressor, it binds directly to Wnt ligands and competes with their ability to activate the frizzled receptors [34]. SFRP1 siRNA treatment in the immortalized, non-transformed human mammary epithelial cell line MCF-10A caused a modest but reproducible increase in luciferase activity in a TCF/LEF activation assay. This activity was further enhanced by addition of Wnt-3A conditioned media [34]. As expression of SFRP1 in breast cancer counteracts Wnt signaling, we proposed that its reduction leads to enhanced Wnt signaling in breast cancer cells.

In MDA-MB-231^EpCAM ^cells mRNAs for both SFRP1 and TCF7L2 were down-regulated compared to the corresponding empty vector line and Wnt signaling was about 20% more active. Of note, Low SFRP1 expression in human breast cancer has been reported by several groups independently [35,36]. Both low SFRP1 and TCF7L2 levels have been described in a subset of breast carcinomas derived from infiltrating ductal carcinoma [32]. However, reduction of SFRP1 expression only, was not sufficient to enhance Wnt signaling in Hs578T^EpCAM ^cells compared to Hs578T^control ^cells. Probably, the reduction of both SFRP1 and TCF7L2 is required to more potently enhance Wnt signaling in breast cancer cell lines, and a contribution of additional factors cannot be excluded.

The transcription factor TCF7L2 belongs to the TCF/LEF (T-cell factor/lymphoid enhancer factor) protein family and is expressed in many isoformal variants which can coexist in one cell. The short isoforms lack the C-terminal protein binding sites (CtBP) which convert the long variants of TCF7L2 into a repressor [37]. It is therefore conceivable that downregulation of a repressive variant of the TCF7L2 protein in MDA-MB-231^EpCAM ^cells might favour Wnt signaling mediated through other activating TCF/LEF proteins.

Although downregulation of tumour suppressors is a common mechanism during cancer progression, activation of Wnt signaling via the suppression of repressors observed in our breast cancer cells differs from the mechanism discovered for colon cancer, where Wnt pathway activation occurs through loss-of-function mutations of negative pathway components e.g. adenomatous polyposis coli (APC) or gain-of-function mutations in genes activating Wnt signaling (i.e. β-catenin) [38]. Our findings in human breast cancer lines underscore the complexity of Wnt pathway in human cancer [33].

EpCAM and β-catenin proteins were detectable in nuclear lysates of both MDA-MB-231^EpCAM ^and Hs578T^EpCAM^, but the nuclear staining for EpCAM was negative in confocal microscopy. As the antibody used for microscopical analysis was directed against the extracellular domain, a detection of the shedded cytoplasmic fragment EpICD was not possible. Regarding the results otained for full-length EpCAM in the fractionation experiment, we suppose that EpCAM has a perinuclear location and cofractionates with nuclear proteins. However, further experiments are required to establish a relationship to the perinuclear space and the endoplasmatic reticulum.

The activation of TCF/LEF by β-catenin is a crucial step in the direct activation of Wnt target genes. In many studies the expression of key target genes such as cyclin D and c-myc is used as a read-out for Wnt signaling activity [10,39]. Upregulation of cyclin D and c-myc has been found to be strongly associated with breast cancer progression [38-40]. However, although expressed at moderate levels in parental cell lines, no significant upregulation of both genes could be detected in Hs578T^EpCAM ^and MDA-MB-231^EpCAM ^cells (data not shown). In line with our observation, it has been reported that in the absence of distinct TCF7L2 variants the regulation of common TCF/LEF target genes such as cyclin D1, c-myc, MMP7 and c-jun is not detectable in renal cell carcinoma and colorectal cancer cell lines [41]. As each cell type is able to express multiple isoforms of TCF7L2 the combination of long repressory and short activating variants of TCF7L2 adds even more complexity to the signaling events, thus target gene expression can be affected in many different ways [37,42]. Furthermore, the type of trigger and feedback loops within the Wnt pathway have been shown to shape the gene expression pattern and thus the cellular response in normal and transformed epithelial cells [43]. Of note, Western blot analysis of MDA-MB-231^EpCAM ^cells showed reduced levels of both short and long TCF7L2 variants.

The role of ITF-2, which is strongly down-regulated in MDA-MB-231^EpCAM ^cells, remains unclear. ITF-2 is a basic-helix-loop-helix (bHLH) transcription factor. Its interaction with the inhibitors of DNA binding Id-1 and Id-2 proteins has been described. In complex with Id proteins, ITF-2 can modulate the mammary epithelial cell phenotype and malignant transformation [44]. Zhai et al showed an interference between ITF-2 and Wnt signaling [45]. Furthermore, the appearance of two isoformal variants, ITF-2A and ITF-2B, differing substantially in domain architecture and function, has been reported [30].

Finally, an increase in invasion and migration after EpCAM overexpression was not observed. Such complex processes are governed by multiple molecular changes. The addition of a single gene might not be sufficient and essential interaction partners might be absent in our analysed cell lines. As such, EpCAM has been described to be a transcriptional target of p53 [46]. A loss of wild-type p53 during breast cancer progression does probably not result in a sole increase of EpCAM expression but also in the regulation of other important target genes.

## Conclusions

The effects of constitutive expression of the EpCAM oncogene are dependent on the properties of parental cell lines. Further, the functional alterations observed *in vitro *were marginal, potentially due to the lack of accessory molecules which might be necessary for EpCAM to exert its full oncogenic potential. In MDA-MB-231^EpCAM ^cells a nuclear accumulation of ß-catenin and a significant upregulation of Wnt reporter assay activity were observed. In Hs578T^EpCAM ^cells, overexpression was accompanied by a considerable decreased expression of the Wnt pathway inhibitor SFRP1, by an increased proliferation rate and enhanced sensitivity to Docetaxel. Certainly, future investigations are warranted to define the functional relationship between increase of EpCAM expression, Wnt signaling and oncogenic features.

## List of abbreviations

bHLH: (basic-helix-loop-helix); CAM: (cellular adhesion molecule); c-myc: (v-myc myelocytomatosis viral oncogene homolog); E-FABP: (epidermal fatty acid binding protein); EpCAM: (epithelial cell adhesion molecule); EpICD: (EpCAM intracellular domain); FHL2: (four and a half LIM-only protein 2); SFRP: (secreted frizzeled related protein); GAPDH: (glyceraldehyde-3-phosphate dehydrogenase); IRES: (internal ribosome entry site); LEF: (lymphoid enhancer binding factor); SDS-PAGE: (sodium dodecylsulfate polyacrylamide gel electrophoresis); Wnt pathway (wingless-type MMTV integration site family signaling pathway)

## Competing interests

The authors declare that they have no competing interests.

## Authors' contributions

JMG established the stably transfected cell lines, carried out migration assays and TCF/LEF reporter assays and contributed substantially in the experimental design. FL and DF carried out Western blots and cell fractionation assays. AM carried out chemosensitivity assay. OAW performed array data analysis. MZ performed PCR analysis. MH carried out immunofluorescence imaging. SK participated in the design of the study. GS and GG conceived the study, and participated in its design and coordination and drafted the manuscript. All authors read and approved the final manuscript.

## Pre-publication history

The pre-publication history for this paper can be accessed here:

http://www.biomedcentral.com/1471-2407/11/45/prepub
